# Medical Anecdotes

**Published:** 1804-11-01

**Authors:** 


### MEDICAL ANECDOTES.

ANECDOTES, relating to history or biography, have ever been thought instructive, as well as amusing; that medical anecdotes would be well received by the profession, few will doubt; how far the following (which may be succeeded by others) are deserving of being preserved in the Medical and Physical Journal, the enlightened Editors will determine.

### *I. Fees of Medical Men.*

E. Maynwaring, doctor in physick, in his Praxis Medicorum antiqua et nova, 1671, says, "Now imagine we were at a file of bills." [prescriptions.] This *recipe* cost the patient *ten shillings* fee, because he was but a doctor of *little* practice, not *cryed* up, and that was fair for him.

Here's another cost a *guiny*; this was a *great doctor's*, one of the eminentest in the town, a man of very *great practice*, that you must wait two hours before you can speak with him, except you give his man a couple of shillings; this must needs be an able man, that the people croud after, much spoken of, and much approved by his *apothecary*, who gets four or five hundred pounds *per annum* by this *doctor's* practice; an excellent *apothecary doctor!* he deserved a piece very gallantly. But here is a *recipe* cost 3 *guynies*; this was the result of a *consultation*, for a person of quality, a beloved child, wife, or husband, or some rich fellow that would *die* more *honourably* than ever he had lived."

Robert Godfrey, Medicus Londinensis, in his "Various Injuries and Abuses in Chymical and Galenical Physick," 1674, tells of a "rich physician," who was employed to attend a poor washerwoman, and received a fee of only 2s. 6d. for each visit. "The doctor visited her once, and had one half-crown, which was more than she could clear by a week's washing; the second day he came again, without sending for, for the doctor's custom was, being once sent for, to follow his game close, and then he had the second half crown; and the third day, when he called in without sending for, the poor woman's last half-crown being hard to be parted with, she did not give him it; whereupon, being angry, he asked her at his departure, whether she thought he could run up and down for nothing?"

"Van Helmont tells you, in the words of the wise man, th that a physician shall receive a gift of a king not of a poor man; thereby implying, that we are not to neglect the poor, though they are not able to bring *angels*, nor crowns, in their hands, for scribbling a few words to an apothecary."

Godfrey, in several places of his book, speaks of an *angel* as being the usual fee of a physician, yet it appears, from what has been mentioned above, that they were contented sometimes to take less.

Advertisement.—At the Angel and Crown, in Basing Lane, near Bow Lane, lives J. Pickey, a graduate in the University of Oxford, and of many years standing in the College of Physicians, London; where all sick people, that come to him, may have for 6d. a faithful account of their diseases, and plain directions for diet, and other things they can prepare themselves; and such as have occasion for medicines may have them of him, at reasonable rates, without paying any thing for advice; and he will visit any sick person in London and the liberties thereof, in the day time, for 2s. 6d. and any where else within the bills of mortality for 5s.; and if he be called for any person, as he passes by, in any of these places, he will require only 1s. for his advice.

*Postman, Jan.* 16, 1700.

An anonymous writer, in 1702, says, "I have known an apothecary make fifteen pounds of a patient in ten days time, by rating the boluses at 2s. 6d. a piece, and other medicines proportionably."

The same author complains, that if a physician ordered an electuary of four ounces, the apothecary would divide it into twenty or thirty boluses, at 1s. 6d. each, and a quart apozem into four half pint phials, each charged 3s. or 3s. 6d.

Another anonymous physician, in 1669, says, "We many times prescribe a drachm of treacle, worth two pence, to a poor neighbour, out of charity; the apothecary makes him pay half a crown for a cordial bolus. There are of us, have retrieved some of our prescriptions, and the apothecaries' bills upon them; you will perhaps be amazed when I tell you, that where a physician hath, without a fee, prescribed something worth sixpence, because it was made into twenty-four pills, there was so many shillings paid to the apothecary upon his bill for it."

The usual charge for draughts at this time was 1s. or 1s. 6d. each; clysters 2s. 6d. and other things in proportion, tion, which is infinitely more, considering the difference in the value of money, than is charged in London at present. One practitioner is mentioned to have "bragged, that he made from 20 to 100l. commonly, *besides presents*, for the cure of a gonorrhœa.

*II. Apothecaries intrenching on the Physician's Province, by visiting and prescribing to Patients.*

"The next thing to be treated of, shall be the ways of apothecaries creeping into practice. Heretofore when they were members of the company of grocers, and dispersed in place, as well as in counsel, they then were wholly subordinate to the physicians, only keeping in their shops, and faithfully making the prescriptions they received from the physician; and when made, sending them to the patient by their men, (as they still continue to do in foreign countries.) But in process of time, physicians, in acute diseases, having taught them somewhat, sent them to visit their patients, to give them the best account they could of the state of their health, and effect of their medicines. And of later years, some physicians took them along with them in their visits, whereby they acquired a little smattering of diseases, by which means they made people believe they had acquired some skill in the art, and afterwards began to venture a little at practice, and, but until these 10 years last past, kept themselves within some bounds and limits; but since that time, have daily more and more encroached upon our profession." 1669.

"In the plague time, [1666] (most physicians being out of town,) they took upon them the whole practice of physic, which ever since they have continued, being much helped also therein, by the dispersion of physicians into places unknown to their patients, by the fire."

Murett's Short View of Frauds and Abuses, 1669.

*III. Frauds of Apothecaries.*

Dr. Murett, in the above pamphlet, charges the apothecaries with "falsifying of medicines. First, they use medicines quite contrary to the prescription; myrtle leaves shewed the Censors for sena, a binder for a purger, mushrooms rubbed over with chalk for agaric, hemlock dropwort roots for pæony roots. Privet by some, by others dog-berries, for those of spina cervina; no purgers for a strong one; *sheep's lungs for fox lungs, the bone of an ox's heart for that of a stag's heart,* damsons for damasc prunes, syrup syrup of lemons for that of citrons, bryony roots for mechoacan, &c.

### IV. *Number of Apothecaries in London.*

"Hamburgh (as Dr. Pitt's book informs us) has but one apothecary's shop; Stockolm and Copenhagen but four or five a piece; and Paris itself but one and fifty; whereas, in London and the suburbs, we have near a thousand." 1702.

"Mr. Goodwin said, he desired the Censors of the College may demonstrate to this honourable house, out of above 1000 apothecaries shops in London, and seven miles circuit, how many they have destroyed in so public a manner.

Brief for James Goodwin, Chemist and Apothecary, upon his Petition to the House of Lords. 1731.

